# Determinants of the use of insecticide-treated bed nets on islands of pre- and post-malaria elimination: an application of the health belief model in Vanuatu

**DOI:** 10.1186/1475-2875-13-441

**Published:** 2014-11-20

**Authors:** Noriko Watanabe, Akira Kaneko, Sam Yamar, Hope Leodoro, George Taleo, Takeo Tanihata, J Koji Lum, Peter S Larson

**Affiliations:** Department of Parasitology, Osaka City University Graduate School of Medicine, Osaka, Japan; Department of Microbiology, Tumor and Cell Biology, Karolinska Institutet, Stockholm, Sweden; Ministry of Health, Port Vila, Vanuatu; Department of Infectious Disease Control, Healthcare Center of Kobe, Kobe, Japan; Departments of Anthropology and Biological Sciences, Binghamton University, Binghamton, NY USA; Nagasaki University Institute of Tropical Medicine, Nagasaki, Japan; University of Michigan School of Natural Resources and Environment, 440 Church Street, Ann Arbor, MI USA

**Keywords:** Malaria, Insecticide-treated net (ITN), The Health Belief Model (HBM), Motivation, Sustainability, Malaria elimination, Islands, Aneityum, Ambae

## Abstract

**Background:**

Insecticide-treated nets (ITNs) are an integral piece of any malaria elimination strategy, but compliance remains a challenge and determinants of use vary by location and context. The Health Belief Model (HBM) is a tool to explore perceptions and beliefs about malaria and ITN use. Insights from the model can be used to increase coverage to control malaria transmission in island contexts.

**Methods:**

A mixed methods study consisting of a questionnaire and interviews was carried out in July 2012 on two islands of Vanuatu: Ambae Island where malaria transmission continues to occur at low levels, and Aneityum Island, where an elimination programme initiated in 1991 has halted transmission for several years.

**Results:**

For most HBM constructs, no significant difference was found in the findings between the two islands: the fear of malaria (99%), severity of malaria (55%), malaria-prevention benefits of ITN use (79%) and willingness to use ITNs (93%). ITN use the previous night on Aneityum (73%) was higher than that on Ambae (68%) though not statistically significant. Results from interviews and group discussions showed that participants on Ambae tended to believe that risk was low due to the perceived absence of malaria, while participants on Aneityum believed that they were still at risk despite the long absence of malaria. On both islands, seasonal variation in perceived risk, thermal discomfort, costs of replacing nets, a lack of money, a lack of nets, nets in poor condition and the inconvenience of hanging had negative influences, while free mass distribution with awareness campaigns and the malaria-prevention benefits had positive influences on ITN use.

**Conclusions:**

The results on Ambae highlight the challenges of motivating communities to engage in elimination efforts when transmission continues to occur, while the results from Aneityum suggest the possibility of continued compliance to malaria elimination efforts given the threat of resurgence. Where a high degree of community engagement is possible, malaria elimination programmes may prove successful.

**Electronic supplementary material:**

The online version of this article (doi:10.1186/1475-2875-13-441) contains supplementary material, which is available to authorized users.

## Background

An estimated 3.3 million lives have been saved since 2000 as a result of a major scale-up of vector control interventions, including high coverage of insecticide-treated nets (ITNs) through combined catch-up (mass, free distribution of ITNs) and keep-up (long-term, routine access to new ITNs) strategies despite the global funding gap [1, 2].

Various factors are associated with the use and non-use of ITNs in malaria endemic areas [3–13]. Free and comprehensive ITN distribution programs may successfully increase the level of ITN ownership and encourage high levels of coverage compensating for a lack of household or administrative financial resources [3–5, 10–13]. However, resource challenged and undeveloped infrastructure is associated with decreased likelihood of both ITN ownership and use even when ITNs are possessed [6, 8, 9, 13]. Free ITN programs may induce a community wide expectation of free ITNs though some programs have been criticized for enabling the diversion of ITNs for uses other than malaria control in extremely poor settings [6, 9, 12]. Common factors that may predict consistent ITN use in Papua New Guinea [11], Solomon Islands [3, 12] and Vanuatu [4] are knowledge [3, 4, 11, 12], malaria risk perception [3, 4, 11], social life (activities, sleeping place and hanging space) [3, 4, 11] and ITN accessibility, sufficiency, price, physical condition, maintenance, replacement, effectiveness and insecticide [3, 4, 11, 12]. Common factors that may cause variation in ITN use and compliance are seasonal factors such as heat, mosquito density and variable levels of transmission [3, 4, 11, 12].

This paper aims to investigate the perceptions and beliefs about malaria and the use of ITNs in Vanuatu (a country on the verge of malaria elimination [14]). Vanuatu is an archipelago of 83 islands located in the southwest Pacific. The vast majority of ni-Vanuatu, as the indigenous population is known, live in rural areas and engage almost wholly in subsistence farming [15]. *Plasmodium falciparum* and *Plasmodium vivax* transmission persist throughout the majority of Vanuatu’s islands, while *Plasmodium malariae* transmission rarely occurs [1, 16]. Vanuatu has two seasons: the cold, dry season from May to October, and a hot, wet (rainy) season from November to April. *Plasmodium falciparum* incidence shows marked seasonality, whereas *P. vivax* incidence shows less marked seasonal patterns [16–18]. In 2008, Vanuatu formally declared a national goal of eliminating malaria by 2020 using a spatially progressive strategy with significant financial support being made available mainly through the Global Fund to fight AIDS, Tuberculosis and Malaria [14, 19]. In cooperation with efforts to eliminate lymphatic filariasis, a national ITN programme has resulted in a sharp decline in malaria cases [17, 20]. The Malaria Indicator Survey (MIS) in 2011 indicated that 71.9% of surveyed people slept under an ITN the previous night, given that the household owned at least one ITN. The MIS also found that Vanuatu households owned an average of 1.99 ITNs per household [19]. Likely as a result, the annual parasite incidence (API) decreased from 73 per 1,000 population in 2003 to nine per 1,000 population in 2011 [21]. As the burden of malaria decreases, it will be important to understand the perceptions of preventive measures to sustain gains in the control and eventual elimination of disease [22].

As ITN programmes depend on the acceptance and active involvement of individuals and communities, human behavioural and social factors will influence ITN use [13]. The Health Belief Model (HBM), a framework commonly used to explore compliance to health interventions [23–25] including community-based interventions [24] can be used to interpret perceptions and net-use behaviours as was shown in previous studies in Tanzania [5, 7]. The HBM has six constructs to explain and predict preventive health behaviours: modifying factors, perceived threat (severity and susceptibility), benefits, barriers, self-efficacy and cues to action [23–25]. In this study, the HBM framework was used to explore and predict health behaviours (consistent ITN use) in the context of reduced malaria risk.

## Methods

### (i) Study settings and design

**Figure 1:**
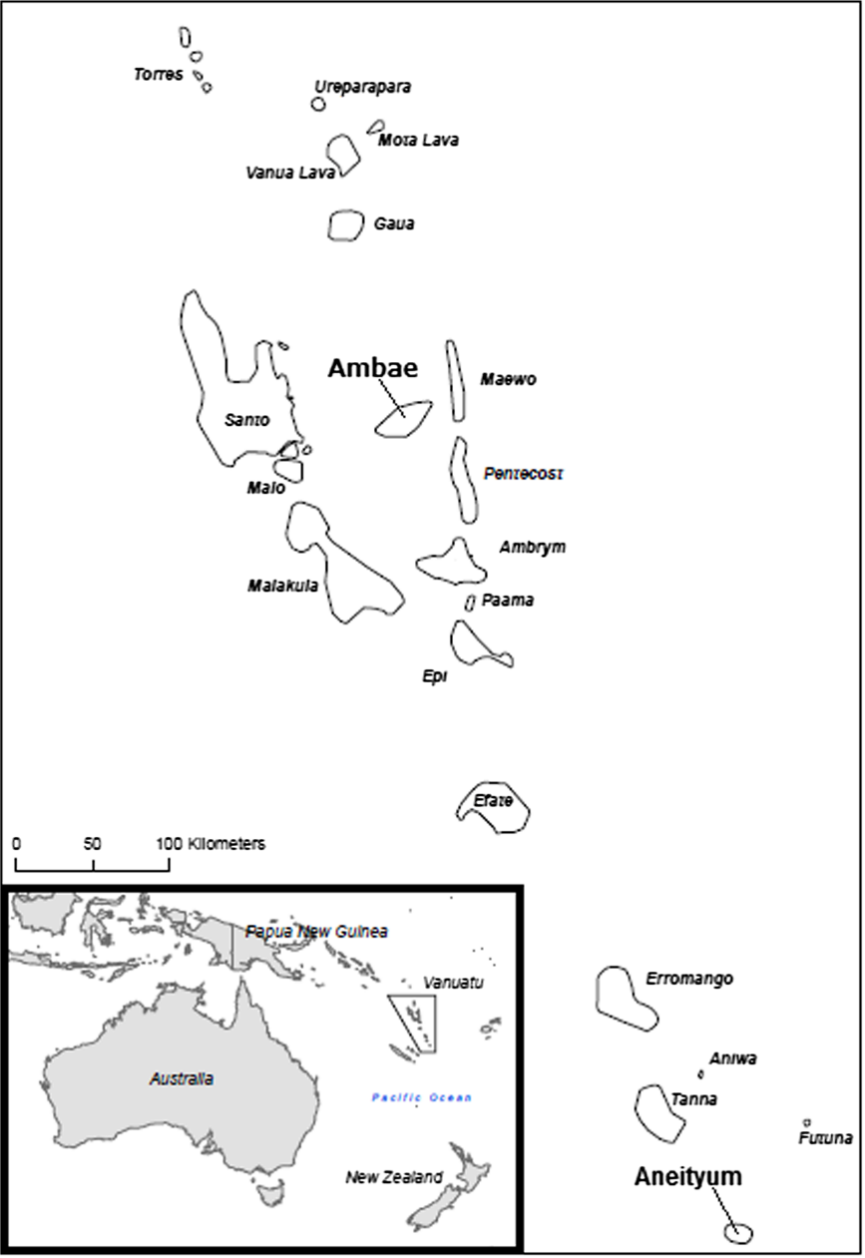
**Locations of study areas on Vanuatu.**

The study areas were purposefully selected to contrast a region where malaria transmission continues to occur with an island that has sustained elimination for several years. Ambae Island, an area of low and sporadic malaria transmission, is located in Penama Province. Aneityum Island, where a community-based, elimination-specific effort since 1991 has successfully halted malaria incidence, is located in Tafea Province in the south [26–28] (Figure 1).

### Ambae

Transmission of *P. falciparum, P. vivax* and *P. malariae* in the area is considered meso-endemic [16]. Indigenous malaria transmission persists, although the prevalence rate of malaria has been declining in recent years (Kaneko *et al.,* unpublished data). Ambae island (405 sq km) has a population of 10,407 (2009 Vanuatu National Census). The study was conducted at Lolovoli village in northeast Ambae with a population of over 200.

### Aneityum

Transmission of *P. falciparum and P. vivax* in the area was considered hypo- to meso-endemic before the introduction of an elimination-specific intervention [16, 26]. In 1991, weekly mass drug administration (MDA) with chloroquine, pyrimethamine/sulphadoxine and primaquine to the entire population (718 islanders) was carried out on Aneityum Island for nine weeks [26, 27]. Simultaneously, ITNs were provided to the entire population, free of charge to mothers and children less than five years of age, and at a cost of US$4 (real price) to adults and US$2 to schoolchildren [26, 27]. In addition, larvivorous fish were introduced into breeding sites of *Anopheles farauti*[26, 27]. After the MDA, *P. falciparum* immediately disappeared, while *P. vivax* disappeared from 1996, with the exception of two instances of imported infections (one mixed infection in 1993 and one *P. vivax* infection in 1999) [26, 27]. In 2002, a small outbreak of *P. vivax* was reported mainly among children born after 1991 [27, 28]. After the age-selected MDA using chloroquine and primaquine, the outbreak quickly subsided except for a few asymptomatic infections of *P. vivax*[27].

Aneityum Island has a total land area of 159.2 sq km with a population of 915 (2009 Vanuatu National Census). Inyeug has a small airport and grass runway. Tourism is the main source of income. There are three main villages: Analgaut, Port Patrick and Unmet [26, 27]. The study was conducted at the largest village, Analgaut, due to its population size and ease of access.

### (ii) Data collection procedures

Data were collected on the two islands in July 2012. Residents were notified ahead of time by local leaders and health workers. Local leaders called a community meeting and explained the intent and process of the survey. At that time, community members were asked to take part in the survey and requested to arrive at the dispensary or community meeting place on scheduled dates.

### Knowledge, attitudes and practices (KAP) survey

The team conducted a cross-sectional survey (knowledge, attitudes and practices) on the two islands. A questionnaire (see Additional file 1) translated into Bislama was administered by local survey assistants fluent in local languages and known to the community and who had been hired and trained to interview respondents. Responses for very young children were provided by an accompanying parent or adult household representative. A questionnaire was structured to capture some of the HBM constructs (perceived severity, benefits and self-efficacy) and action (ITN use the previous night). Potential answers (reasons) for non-use such as absence of mosquitoes/rain, low risk of infection (not being afraid of contracting malaria), excessive heat, inconvenience of hanging nets, nets in poor condition and a lack of nets in the home were provided in a questionnaire.

### Interviews (FGDs, KIIs and IDIs)

For data triangulation, focus group discussions (FGDs), key informant interviews (KIIs) and in-depth interviews (IDIs) were used. A researcher with local facilitators, conducted all discussion and interviews on both islands. Focus group discussants were recruited by local facilitators. Key informants such as *kastomary* chiefs (customary chiefs), teachers, religious leaders, health committee members, health care workers, and shop sellers were purposefully selected. In-depth interviewees were recruited through convenience sampling to ensure a breadth and depth of insights.

Semi-structured interview questions (see Additional file 2) were pilot tested on a few informants to review the answers and assess the relevance to the different HBM constructs. All FGDs, KIIs and IDIs were recorded using notes and a digital device in the presence of local facilitators. The questions were asked in English by a researcher, and then translated into Bislama by local facilitators. The participants’ answers were directly translated from local language to English by local facilitators. Where this was not necessary, English was used as a common language. Each interview transcript was shared and discussed among local facilitators and key informants to explain results and obtain feedback.

### (iii) Data management and analysis

#### Analysis of the KAP survey instrument (statistical methods)

Differences between the two islands were analysed using standard Chi-square tests for categorical variables. T-tests and Wilcoxon tests were used to compare continuous measures between islands. All statistical analyses were performed using R version 2.15.1 (CRAN 2012).

#### Analysis of interviews

The HBM was used as the theoretical framework, where six main HBM constructs (i.e., severity of malaria, susceptibility to malaria, benefits of ITN use, barriers to ITN use, cues to ITN use and self-efficacy) served as the pre-existing categories. The theory-based analysis style (a deductive approach) was applied [29, 30]. First, a categorization matrix was developed [30]. Each interview transcript was read multiple times to identify meaning units, which were condensed, coded and assigned to the pre-existing HBM categories in a matrix [30, 31].

#### Parallel analysis in a mixed methods study

This study employed parallel analysis in a mixed methods study [32, 33]. Data collection and analysis were carried out separately and the findings were not compared or consolidated until the interpretation stage [33]. Qualitative and quantitative results were used to complement each other [29, 33].

#### Ethical considerations

This research was approved by the Vanuatu Ministry of Health, and the Institutional Review Board of State University of New York, Binghamton (#1578-10). Written and verbal consent was obtained before starting the survey and interviews. All respondents were assured that their responses would remain confidential.

## Results

### KAP survey

The result from the KAP survey instrument used on Ambae and Aneityum islands are presented in Table 1. A total of 91 of nearly 200 residents participated in the survey in the village of Lolovoli on Ambae Island, compared to 354 of nearly 400 residents of Analgaut village on Aneityum Island. Educational attainment of adults (aged 18 or older) was similar in both islands. The vast majority of adults had completed only primary education, with half completing secondary school (modifying factors). About half of the residents of Ambae, but only one quarter of Aneityum (48.8 *vs* 25.3%, P <0.0001), reported having been diagnosed with malaria by a health professional in the past (modifying factors). Significantly more children under the age of five on Ambae reported having had malaria in the past than on Aneityum (42.9 *vs* 7.9%).

**Table 1:** **Knowledge, attitudes and practices (KAP) survey results**

HBM constructs	Questions	All	Ambae	Aneityum	P
	N	445	91	354	
	Under 5 years old	53	6	47	
	5 to 17 years old	215	34	181	
	18 to 30 years old	69	15	54	
	Over 30 years old	108	36	72	
	Age range	0-75	0-75	0-74	
	Female	221	49	172	
**Action**	Used last night	72.0%	68.2%	73.0%	0.44
**Modifying factors**					
Educational attainment (aged 18 or older)	None	0.6%	0.0%	0.8%	0.22
	Primary	41.2%	45.1%	39.7%	
	Secondary	48.6%	47.1%	49.2%	
	Unknown	9.6%	7.8%	10.3%	
Malaria history	I had malaria	30.1%	48.8%	25.3%	<.0001
By age	Under 5 years old	13.3%	42.9%	7.9%	
	5 to 17 years old	20.1%	35.7%	17.5%	
	18 to 30 years old	32.8%	50.0%	27.7%	
	Over 30 years old	54.2%	60.0%	51.4%	
**Individual beliefs**					
Perceived severity	I am afraid of malaria (disease)	98.7%	100%	98.4%	1
	Malaria is deadly	55.1%	55.3%	55.1%	1
Perceived benefits	Malaria-prevention	79.4%	78.8%	79.6%	1
Self-efficacy	I am willing to sleep under a net	92.8%	95.3%	92.2%	0.4

No significant difference was found between the two islands in terms of questions which might be applicable to the HBM constructs (perceived severity, benefits, and self-efficacy) and action. ITN use the previous night on Aneityum (73.0%) was higher than that on Ambae (68.2%) although not significant. Nearly all survey respondents claimed to be afraid of malaria (severity). Half stated that malaria was a deadly disease (severity). Seventy-nine per cent of survey respondents reported that they considered ITNs to be an effective means of malaria prevention (the malaria-prevention benefits). 93% of respondents reported liking to sleep under an ITN (self-efficacy).

**Figure 2:**
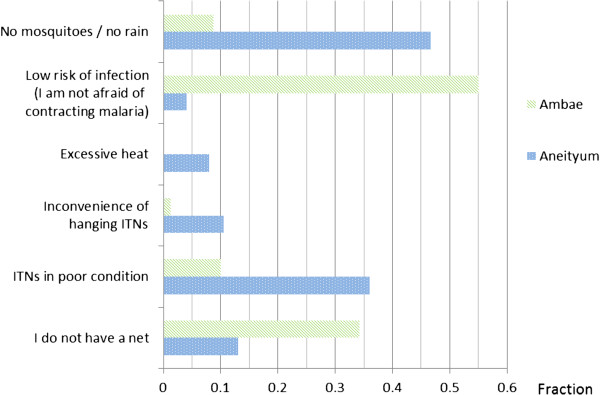
**Reasons for non-use of insecticide-treated bed nets based on knowledge, attitudes and practices (KAP) survey.**

The reasons given for a lack of ITN use are shown in Figure 2. Although the malaria history of respondents under the age of five suggested indigenous malaria transmission on Ambae during recent years (Table 1), low malaria risk perception was the most common barrier to compliance (Figure 2). On Aneityum, perceived low mosquito density with excessive heat acted as barriers to ITN use during the dry season (Figure 2). On both islands, a lack of nets in the home, poor condition and the inconvenience of hanging were common barriers (Figure 2).

### Interviews

**Figure 3:**
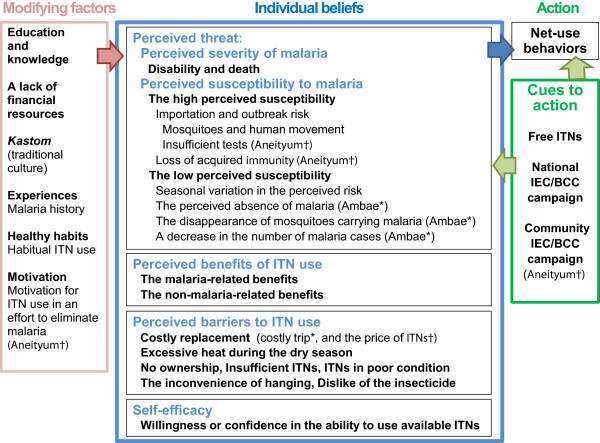
**Results from interviews.**

A total of 35 residents of Lolovoli village on Ambae Island took part in five FGDs, seven KIIs and eight IDIs, while a total of 57 residents of Analgaut on Aneityum took part in six FGDs, ten KIIs and 17 IDIs on Aneityum (Table 2). All results are presented in Figure 3.

**Table 2:** **Demographics of interviews**

Ambae			
	N	Age (years old)	Female
**FGD**	20 (5 groups)	13-44	65.0%
**KII**	7	28-64	20.0%
**IDI**	8	20-52	40.0%
**ALL**	35	13-64	54.3%
**Aneityum**			
	**N**	**Age (years old)**	**Female**
**FGD**	30 (6 groups)	16-31	66.7%
**KII**	10	21-65	50.0%
**IDI**	17	16-67	52.9%
**ALL**	57	16-67	59.6%

FGD: Focus Group Discussion.

KII: Key Informant Interview.

IDI: In-Depth Interview.

### Action

Many participants on both islands reported using ITNs. A few key informants on both islands stated that they had screens on windows. A few households on both islands reported not owning an ITN. Some participants on both islands noted that their ITNs had multiple holes. A few participants on both islands noted that they used their ITNs despite having multiple holes. A few participants on both islands reported alternative use.

*“A few people recycled old nets. Pieces were used for various purposes.” (Male FGD, Ambae)*

*“Five years ago, I saw a child using nets for fishing. Now, health committee encourages people not to use nets for farming or fishing.” (Female KII, Ambae)*

*“I saw some children using nets for fishing this year.” (Male KII, Aneityum)*

A male key informant on Aneityum expressed his concern over the ecological and safe disposal methods for used and worn-out ITNs.

### Modifying factors

A few participants on both islands noted that they did not know how to read and write. All participants were aware that mosquito bites are associated with malaria. Some participants on both islands stated that they had financial difficulty in paying school expenses for several children. *Kastom* (traditional) medicine was used on both islands. A few adults from both islands who reported having had malaria in the past stated that they used ITNs. Some participants on both islands reported forming proactive health behaviours.

*“Some children use nets. They are used to sleeping under a net.” (Female KII, Ambae)*

*“I use a net every day. I am used to sleeping under a net, because I try to prevent malaria.” (Male IDI, Ambae)*

*“I use a net. Everyone uses a net. We feel strange, if we do not use nets.” (Female IDI, Aneityum)*

Participants on Ambae were reported difficulties in consistently using ITNs, while participants on Aneityum reported being highly motivated to use them. On Ambae, many participants stated that the disappearance of malaria reduced the perceived need for sleeping under ITNs. In this context, some male discussants and key informants expressed difficulties in sustaining malaria control efforts in the village.

*“It is very difficult to sustain efforts, because of the absence of malaria. Some people do not use nets. After the net distribution, malaria is not endemic.” (Male FGD, Ambae)*

*“Malaria is disappearing. Some people no longer need nets. Raising awareness is necessary, but a community radio has been broken. Additional funding will be needed to repair a radio. This area faces critical shortage of nurses. The dispensary and hospital are too far. This is a completely neglected area. We need more funds.” (Male KII, Ambae)*

In contrast, an effort to promote healthy life and to eliminate malaria on Aneityum motivated many individuals to use ITNs. Most people reportedly engaged in elimination efforts.

*“We are happy to live on this island. We try to maintain a healthy environment. We sleep under a net and keep our village clean.” (Female FGD, Aneityum)*

*“Malaria depopulated this island in the past. Now we should increase the population. Many people use nets to live a healthier life.” (Male KII, Aneityum)*

### Perceived threat of malaria (perceived severity of and susceptibility to malaria)

Participants on Ambae were likely to state that some villagers did not use ITNs because they were not concerned about the possibility of contracting malaria, while participants on Aneityum stated that using ITNs was necessary out of fear that accidental importation could cause an outbreak of malaria on the island.

Malaria was not recognized as a matter of community concern requiring community action on Ambae. A few youth interviewees stated that visitors from the malarious villages would drive parasite importation, while many participants believed that they were not at risk for malaria infection. Beliefs about the disappearance of malaria transmitting mosquitoes and the reduction in malaria cases were cited as major reasons for decreased concerns of risk.

*“Last year, Lolowai hospital malaria team came and killed mosquito larvae. Now we are not bothered by malaria-infected mosquitoes. All 14 patients with fever had negative RDT results in this year. Men were not willing to come to the aid post at the onset of fever. No new case of filariasis, dengue fever, or malaria has occurred. But some people keep on using nets, because they are afraid of malaria, and they are used to sleeping under a net.” (Female KII, Ambae)*

*“We live in a relatively cold climate on the island. The cold weather reduces mosquito populations. Other villages have more mosquitoes. People catch malaria at South Ambae or North Ambae, but no one has malaria in this village, because anopheline mosquitoes are not found in this village. So some people do not use nets. I am usually bitten 20 times per day by mosquitoes such as Culex and Aedes. A few cases of malaria have been reported in recent years. Malaria is not a serious problem in this village. But malaria remains a serious problem in Sakao.” (Male IDI, Ambae)*

In contrast, malaria was widely perceived as a serious disease and recognized as a matter of community concern requiring community action on Aneityum. Except for a few participants who did not perceive themselves to be at risk for malaria infection, most participants on Aneityum believed malaria posed a very real threat.

*“We want live in a healthy environment. We fear that imported malaria will increase. Because only one person takes blood on this island.” (Female KII, Aneityum)*

*“I am afraid of malaria. Malaria will come back again. So, I think I need a net.” (Male IDI, Aneityum)*

Young people were viewed as more susceptible to malaria due to loss of acquired immunity by some participants on Aneityum.

*“Older people would notice the signs of malaria. But younger people would not notice. Their bodies do not learn how to deal with malaria. We used to have malaria here, but we do not have malaria now. I do not know what will happen in the future.” (Female KII, Aneityum)*

Community members on Aneityum were very concerned that both humans and mosquitoes constituted and continuing threat to maintaining malaria elimination.

*“A big swamp has been managed by committee members. Most people use nets every day.” (Female KII, Aneiyum)*

*“The only problem on this island is that malaria-infected people stay without being tested.” (Male KII, Aneityum)*

*“Malaria-carrying mosquitoes will kill us. I use a net.” (Female IDI, Aneityum)*

*“Because Aneityum is a place of quarantine in Vanuatu, many yachts visit this island. Some foreigners walk around the village without the testing for a few weeks. Local boat passengers are not screened. I am worried that I may have contracted malaria in Tanna, because we (fishers) travel back and forth. All people entering Aneityum are not screened. Air passengers have been screened, but cruise passengers have not been screened this year. An airplane and a ship are problematic.” (Male IDI, Aneityum)*

### Perceived benefits of ITN use

The most cited benefit of ITN use on both islands was the prevention of malaria. The protection from mosquito bites was the other most commonly cited benefit on both islands.

*“Malaria-infected anopheline mosquitoes are not found in this village, however people still use nets, because people protect themselves from mosquito bites. I am bitten all the time.” (Female KII, Ambae)*

*“I appreciate the benefits of the insecticide, because the treated nets provide very good protection from being bitten by mosquitoes. I use a net every day.” (Male KII, Aneityum)*

Protection against other diseases (lymphatic filariasis on Ambae, and scabies on Aneityum) as well as against pests (cockroaches, fleas, flies, and head lice on Aneityum), and keeping warm in cold weather on Ambae were very occasionally reported as the non-malaria-related benefits of ITN use. A few key informants on Aneityum noted that mass use of ITNs eliminated scabies.

### Perceived barriers to ITN use and maintenance

On both islands, the most commonly reported barrier to ITN use was that the ITNs were uncomfortable to sleep under during very hot conditions. Many participants reported that they slept without ITNs during the dry season when temperatures are very hot and winds quite weak.

*“When the weather is hot during the dry season, we do not use nets.” (Male KII, Ambae)*

*“If people feel uncomfortable during the dry season, people enjoy sleeping outside and use mosquito coils. If we do not see mosquitoes outside, we do not use coils. During the rainy season, we use nets.” (Female KII, Aneityum)*

Dislike of the insecticide was identified as barriers on both islands by a limited number of participants.

*“I do not use a net, because I use kastom medicine. My grandmother is a kastom healer. But my family members go to the dispensary at the onset of fever. My mother encourages me to go to the dispensary, but I do not want to go.” (Female IDI, Ambae)*

*“I do not like the chemical smell. Babies suck a net. But I use a net, because I know it works for protection.” (Female KII, Aneityum)*

A female discussant on Aneityum noted that she had never slept under an ITN, because she believed that ITNs caused suffocation. A few participants on both islands reported the inconvenience of hanging rectangular ITNs and a preference for conical ITNs.

High costs were reported as barriers to replacement of old and worn out ITNs. Distance and accessibility to ITN distribution points (the cost of travelling to and from hospital in North Ambae) reportedly acted as hurdles to obtaining new ITNs on Ambae. A key informant noted that Lolowai hospital did not allow the aid post to deliver ITNs. The price of ITNs (almost US$5) on Aneityum was noted among the majority of female participants. The issue of user charges was initially raised within the female FGDs. A few discussants and interviewees reported having no intention to purchase in spite of their beliefs about their chances of getting malaria and the malaria-prevention benefits of ITN use. A female interviewee on Aneityum who reportedly did not buy an ITN for her baby stated that she would be willing to get free ITNs. Another female interviewee on Aneityum noted that she sold a portion of her crops or woven baskets to purchase a new ITN.

### Self-efficacy and cues to action

Most participants on both islands stated that they were confident in their ability to use available ITNs. Receipt of a free ITN was commonly seen as beneficial on both islands.

*“In the past, nets were not easily available for every person. Now nets are distributed free of charge. Everyone has a net. That is why people keep and use.” (Male KII, Ambae)*

*“In the past, rich people could afford to buy nets, but now everybody has a treated net. That is good.” (Female KII, Aneityum)*

A few key informants on Ambae stated that high community coverage would contribute to a reduction in malaria transmission.

All participants on both islands recognized the sources of malaria information (cues) such as service delivery points (the dispensary on Aneityum and hospital on Ambae), health staff, the provincial malaria team, public notice, a community meeting, family meeting, health talk, church activities, school activities and Radio Vanuatu. Some male discussants and key informants on Ambae reported that they suffered lack of funds to sustain government-led efforts against a disappearing disease, while most participants on Aneityum intended to utilize available resources to prevent the re-introduction of malaria. Community-based elimination activities such as surveillance, vector control measures and awareness campaigns reportedly encouraged consistent ITN use on Aneityum.

### KAP survey and interviews

Results from the KAP survey (Figure 2) and interviews (Figure 3) showed that the HBM constructs which negatively influenced ITN use were the low perceived susceptibility to malaria (a reduction in malaria risk) and barriers to ITN use (Table 3). The low perceived susceptibility had more impact on Ambae than Aneityum (Table 3). Results from interviews (FGDs, KIIs and IDIs) revealed the motivation and healthy habits were less influenced by the low perceived susceptibility and perceived barriers.

**Table 3:** **Determinants of non-use**

Low perceived susceptibility					Perceived barriers				
Beliefs	Islands		QL	QT	Beliefs	Islands		QL	QT
Dry season	Am	An	QL	QT	Excessive heat	Am	An	QL	
							An		QT
Perceived low mosquito density	Am	An	QL	QT	Dislike of the insecticide	Am	An	QL	
Low risk of infection	Am	An	QL	QT	Inconvenience of hanging nets	Am	An	QL	QT
Perceived absence of malaria	Am		OL		Nets in poor condition	Am	An	QL	QT
Disappearance of mosquitoes carrying malaria	Am		QL		A lack of nets in the home	Am	An	QL	QT
					Costly services				
					Time and cost to access	Am	An	QL	
Reductions in the numbers of malaria cases	Am		QL		User charge		An	QL	

QL: Qualitative results (FGDs, KIIs and IDIs), QT: Quantitative results (KAP survey).

Am: Ambae, An: Aneityum.

This study indicated that three determinants of ITN use influenced net-use behaviours: (1) malaria risk, (2) intervention services (tools and services), and (3) personal factors (modifying factors and self-efficacy) (Table 4). These factors were not independent, but rather were interlinked. Seasonal variation in the perceived risk of malaria influenced utilization of and compliance to malaria interventions and attitudes. Interventions influenced the knowledge, attitudes and practices. A lack of resources such as money, time and knowledge occasionally acted as a brake, while motivational beliefs sustained intervention and malaria risk.

**Table 4:** **Determinants of access and use**

Determinants	Beliefs or factors	HBM constructs	Islands	
**Malaria risk**				
Beliefs or factors associated with ITN access and use	Health consequences (death)	Severity	Am	An
	Social consequences (depopulation)	Severity		An
	Loss of acquired immunity	Susceptibility		An
	Human movement	Susceptibility	Am	An
	Insufficient screening	Susceptibility		An
	Hot, wet (rainy) season	Susceptibility	Am	An
	High mosquito density	Susceptibility	Am	An
	The potential risks of malaria infections	Susceptibility	Am	An
Beliefs or factors associated with non-use	Cold, dry season	Susceptibility	Am	An
	Low mosquito density	Susceptibility	Am	An
	Low risk of infection	Susceptibility	Am	An
	The disappearance of malaria-infected anopheline mosquitoes	Susceptibility	Am	
	The reduction in malaria incidence	Susceptibility	Am	
**Intervention services (tools and services)**				
Beliefs or factors associated with ITN access and use	Free mass distribution (catch-up)	Cues	Am	An
	National campaigns	Cues	Am	An
	Community-based campaigns	Cues		An
	ITN prevention (malaria)	Benefits	Am	An
	ITN protection (mosquitoes)	Benefits	Am	An
	ITN prevention (other diseases)	Benefits	Am	An
	ITN protection (other pests)	Benefits		An
Beliefs or factors associated with non-use	Time and cost to replace nets (keep-up)	Barriers	Am	An
	Insufficient or a lack of ITNs in the home	Barriers	Am	An
	ITNs in poor condition	Barriers	Am	An
	Inconvenience of hanging	Barriers	Am	An
	Excessive heat in the net (discomfort)	Barriers	Am	An
	Side effects of the chemical	Barriers	Am	An
**Personal factors (modifying factors and self-efficacy)**				
Beliefs or factors associated with access and use	Willingness or confidence to use ITNs	Self-efficacy	Am	An
	Knowledge	Modifying factors	Am	An
	Experiences (malaria history)	Modifying factors	Am	An
	Healthy habits (consistent ITN use)	Modifying factors	Am	An
	Motivation for ITN use in an effort to eliminate malaria (healthy life)	Modifying factors		An
Beliefs or factors associated with non-use	Unwillingness to buy or use ITNs	Self-efficacy	Am	An
	The absence of financial resources	Modifying factors	Am	An
	Insufficient knowledge	Modifying factors	Am	An

Am: Ambae, An: Aneityum.

## Discussion

Malaria risk perception (mainly due to perceived susceptibility), free ITNs (cues to action), community-based intervention services (cues to action) and personal factors (modifying factors) encouraged individuals on Aneityum to maintain the use of ITNs, in spite of material and psychological costs. On Ambae, the low perceived susceptibility along with material and psychological costs was associated with reduced compliance to ITNs.

Perceptions of disease severity and the potential risks of malaria infections (importation and outbreak risk [14]) were linked to sustained use of ITNs on Aneityum. However, the perceived absence of malaria was linked to non-use of ITNs on Ambae in spite of a low but still present risk of malaria transmission, suggesting a potentially detrimental effect on sustained ITN coverage and use in the context of disappearing disease. Perceived low mosquito density during the dry season, hampered consistent use of ITNs even on Aneityum. This outcome agrees with previous results on seasonality that may predict variation in ITN use [3–5, 7, 8, 10–13]. Thermal discomfort during the hot, wet season was not mentioned as a barrier to ITN use on both islands, indicating that the perceived higher risk of malaria outweighs the perceived discomfort of being hot during the wet (rainy) season, consistent with a previous study in Zanzibar, Tanzania [5]. Participants did, however, that ITNs were uncomfortable during the dry season, a tendency which could compromise malaria control efforts where malaria transmission still occurs. Psychological, physical or financial burden of services were directly linked to non-use of ITNs on both islands in spite of the malaria-prevention and non-malaria prevention benefits of ITN use. Routine access to new nets (keep-up strategies) had negative influences on ITN ownership on both islands despite free mass distribution (catch-up strategies) and campaigns (cues to actions). Expanding access to keep-up nets may improve coverage and use [2]. As cost significantly dampens demand and decreases access to health services among the poor at greater risk, ITNs should be provided via the public sector as a public good, like vaccines with a generous donation, because malaria is linked to poverty [34–37].

Results from focus group discussions, key informant interviews, and in-depth interviews showed that a lack of resources, discomfort, dislike, unwillingness and insufficient knowledge had negative influences on individual decision making regarding ITN use on both islands, while knowledge of preventive measures against importation and outbreak risk, healthy habits and the motivation had positive influence on decision making on Aneityum. Although a previous study in Burkina Faso has shown that the motivation for the use of new ITNs can decrease within a year due to inhabitants’ conception of malaria and the inconvenience of using ITNs [38], this study showed that the sustained motivation for ITN use in an effort to promote healthy life and to eliminate malaria influenced net-use behaviours on Aneityum, indicating that sustained motivation continuously increases knowledge, encourages healthy habits and creates demand for ITNs regardless of circumstances (local culture, a hot, humid tropical climate, low risk infection, costly services in resource-poor settings and poverty) behind net-use behaviours. This study implies the primary factors that predict high ITN coverage and use on Aneityum are individual and collective motivations and engagement in elimination activities. Unlike the one-off vaccination campaigns, ITN programmes require a high degree of participation and practices to be sustained over time [39]. Community-based campaigns including information, education and communication (IEC)/behavioural change communication (BCC) minimize risk behaviours on Aneityum, where a participatory process maintains high ITN coverage and use at the community level toward malaria elimination. The findings from Aneityum provide clues to sustainable ITN use and malaria elimination in areas of reduced malaria transmission.

### Limitations

The HBM was employed to explore perceptions and beliefs about malaria and ITN use. This study focused on individuals’ beliefs and net-use behaviours without fully taking into account social, economic and emotional factors that may also influence preventive health behaviours. Time was limited and the number of interviews that was feasibly performed was small. In particular, the results from interviews (FGDs, KIIs and IDIs) are limited to be generalized to the wider population of each island. As the survey and interviews were performed during the dry season, the results may have differed during the wet season when mosquitoes are abundant. Some responses relating to ITN use may be subject to social desirability bias. Finally, there may be a certain degree of loss of nuances and depth as a result of the direct translation from Bislama to English in conducting interviews (FGDs, KIIs and IDIs).

## Conclusions

The results on Ambae highlight the challenges of motivating communities to engage in elimination efforts when transmission continues to occur, while the results from Aneityum suggest the possibility of continued compliance to malaria elimination efforts given the threat of resurgence. Where a high degree of community engagement is possible, malaria elimination programmes may prove successful.

## Electronic supplementary material

Additional file 1: **A questionnaire.** (DOCX 34 KB)

Additional file 2: **Interview questions.** (DOCX 28 KB)
