# Supplementary material for: Determinants of the use of insecticide-treated bed nets on islands of pre- and post-malaria elimination: an application of the health belief model in Vanuatu
Source: Malar J. 2014 Nov 20;13:441. doi: 10.1186/1475-2875-13-441 (PMC4289159; doi:10.1186/1475-2875-13-441)
Supplement: Supplementary file 1 — Additional file 1: A questionnaire. (DOCX 34 KB) [file 12936_2014_3647_MOESM1_ESM.docx]

**Additional File 1**

# Determinants of the use of insecticide-treated bed nets on islands of pre- and post-malaria elimination: an application of the health belief model in Vanuatu

Authors: Noriko Watanabe^1^§, Akira Kaneko^1,2^, Sam Yamar^3^, Hope Leodoro^3^ , George Taleo^3^,

Takeo Tanihata^4^, J Koji Lum^5^, Peter S Larson^6,7^

§Corresponding author: Noriko Watanabe: [n881052@gmail.com](mailto:n881052@gmail.com)

This file includes the knowledge, attitudes, and practices (KAP) survey questionnaire.

**INDIVIDUAL QUESTIONS**

- 1. **Knowledge/Attitudes**

1. **What causes malaria?**

1. Mosquitoes 2. Rain 3. Air 4. Devil/Spirits/Magic 5.Other__________

1. **What are the symptoms of malaria (more than one answer is acceptable)?**

| □ a. Fever | □ e. Nausea |
| --- | --- |
| □ b. Chills | □ f. Diarrhea |
| □ c. Headache | □ g. Other _________________ |
| □ d. Dizziness | □ h. Don’t know |

1. **Does sleeping under a net prevent malaria?**

| □ a. Yes | □ d. No |
| --- | --- |

1. **Has anyone ever taught you how to use a net ?**

| □ a. Yes | □ d. No |
| --- | --- |

1. **What is the main purpose of a net?**

a. Keeps insects from biting at night b. Prevents malaria c. Other

1. **Would you like more information on nets?**

| □ a. Yes | □ d. No |
| --- | --- |

1. **Are you afraid of getting malaria?**

| □ a. Yes | □ d. No |
| --- | --- |

1. **Can you die from malaria?**

| □ a. Yes | □ d. No |
| --- | --- |

1. **Is eliminating malaria important?**

| □ a. Yes | □ d. No |
| --- | --- |

1. **If yes, what is the benefit of malaria elimination?**

| □ a. Reduce death | □ d. Tourism |
| --- | --- |
| □ b. Can work more | □ e. Economic growth/jobs |
| □ c. Less money spent on health care | □ g. Other _________ |
| □ d. No benefits | □ h. Don’t know |

1. **If no, why?**

| □ a. There are other diseases/will get sick anyway | □ d. Nothing changes |
| --- | --- |
| □ b. It will come back | □ e. Other _________________ |
| □ c. Don’t care | □ h. Don’t know |

- 1. **Malaria/Net Practice**

1. **What is the most effective way to protect against malaria?**

| □ a. Use ITNs/LLINs | □ e. Burn leaves |
| --- | --- |
| □ b. Indoor spraying (IRS) | □ f. Prayer |
| □ c. Mosquito spray (on body) | □ g. Eliminate breeding sites |
| □ d. Mosquito coils | □ h. Other ________ |
| □ d. Keep house clean | □ h. Nothing |

|  |  |
| --- | --- |

1. **If no, why do you NOT use bed nets? _____________(multiple answer possible)**

| □ a. Condition is bad (holes) | □ e. Too difficult to hang |
| --- | --- |
| □ b. It dry right now | □ f. Don’t have enough nets |
| □ c. No mosquitoes | □ g. Other _________ |
| □ d. Don’t care about malaria | □ h. Don’t know |

1. **Which do you prefer LLIN or ITN?”**

| □ a.LLINs | □ d. ITN |
| --- | --- |

1. **Have you ever participated in malaria education programs?**

| □ a. Yes | □ d. No |
| --- | --- |

1. **Have your habits changed since the program?**

| □ a. Yes | □ d. No |
| --- | --- |

1. **If yes, how so?**

| □ a. Use ITNs/LLINs | □ e. Burn leaves |
| --- | --- |
| □ b. Indoor spraying (IRS) | □ f. Prayer |
| □ c. Mosquito spray (on body) | □ g. Eliminate breeding sites |
| □ d. Mosquito coils | □ h. Other ________ |
| □ d. Keep house clean | □ h. Nothing |

1. **Where do you usually get information/education on malaria?**

| □ a. Health facility/clinic | □ g. Printed material |
| --- | --- |
| □ b. Friends/family | □ h. Radio |
| □ c. Health workers | □ i. TV |
| □ d. Communal gatherings | □ j. Cell phone |
| □ e. Government staff | □ k. School |
| □ f. Church | □ l. God |
|  | □ m. Never get health info |

1. **What kind of information/messages have you gotten in the past?**

| □ a. Malaria is a dangerous disease | □ e. Info on treatment |
| --- | --- |
| □ b. You can die from malaria | □ f. Symptoms of malaria |
| □ c. Info on mosquitoes | □ g. IRS |
| □ d. Bed net info | □ h. Data/statistics |

1. **Of these, which were helpful? ____ (please choose above box)**
2. **In the future, which kinds of information would you like to have?** ____ (please choose the options above)
3. **When you have a health emergency, to whom do you turn to for help?**

| □ a. The closest (proximal) person | □ d. Health workers |
| --- | --- |
| □ b. Friends | □ e. Other ______ |
| □ c. Family | □ f. No one |

- 1. **Health Survey Information (the survey will be announced ahead of time)**

1. **Did you hear about this survey before today?**

| □ a. Yes | □ d. No |
| --- | --- |

1. **If yes, how did you hear about this health survey?**

| □ a. Family/Kin | □ f. Government staff |
| --- | --- |
| □ b. Neighbors | □ g. Health workers |
| □ c. Friends | □ h. Acquaintance |
| □ d. Church people | □ i. Business associate |
| □ e. Teacher |  |
|  |  |

1. **By what means did you hear about this survey?**

| □ a. Cell phone | □ c. Radio |
| --- | --- |
| □ b. In person | □ d. Other _______ |
|  |  |

1. **When did you first hear about this survey?**

| □ a. One week ago | □ c. 2-3 days ago |
| --- | --- |
| □ b. 4-6 days ago | □ d. yesterday |

1. **In the past week, who have you shared meals with (write full names)?**

|  |  |
| --- | --- |
|  |  |
|  |  |
|  |  |
|  |  |
|  |  |
|  |  |
|  |  |
|  |  |
|  |  |

1. **Who do you go to for advice on important matters (full name)?**

**_____________ __________________**

1. **Who do you go to for advice on important health matters (full name) ?**

**______________ _________________**

1. **Did you sleep under a net last night?** □ a. Yes □ b. No
2. **What color was it? ___________________**
3. **Do you like sleeping under a net?** □ a. Yes □ b. No
4. **If no, why?**

| □ a. Too hot | □ c. Too many people |
| --- | --- |
| □ b. Itches | □ d. Other _____________ |

1. **In the past two weeks, have you had diarrhea?** □ a. Yes □ b. No
2. **In the past two weeks, have you had fever?** □ a. Yes □ b. No
3. **In the past, have you ever been told you have malaria by a health professional?**

□ a. Yes □ b. No
