# Supplementary material for: Determinants of the use of insecticide-treated bed nets on islands of pre- and post-malaria elimination: an application of the health belief model in Vanuatu
Source: Malar J. 2014 Nov 20;13:441. doi: 10.1186/1475-2875-13-441 (PMC4289159; doi:10.1186/1475-2875-13-441)
Supplement: Supplementary file 2 — Additional file 2: Interview questions. (DOCX 28 KB) [file 12936_2014_3647_MOESM2_ESM.docx]

**Additional File 2**

Determinants of the use of insecticide-treated bed nets on islands of pre- and post-malaria elimination: an application of the health belief model in Vanuatu

Authors: Noriko Watanabe^1^§, Akira Kaneko^1,2^, Sam Yamar^3^, Hope Leodoro^3^ , George Taleo^3^,

Takeo Tanihata^4^, J Koji Lum^5^, Peter S Larson^6,7^

§Corresponding author: Noriko Watanabe: [n881052@gmail.com](mailto:n881052@gmail.com)

This file includes interview questions.

**Interview questions**

**A research question:**

To identify the beliefs or factors affecting ITN use on the islands of Ambae and Aneityum

**Focus Group Discussions (FGDs)**

1. **ITN ownership and replacement**
2. **The Use of ITNs**

The motivation for the use

The purpose of use

Reasons for non-use

1. **Perceived threat of malaria (severity and susceptibility)**

Malaria transmission

Malaria risk perception

1. **Perceived benefits of ITN use and barriers to ITN use**

Good and bad points of nets

1. **Self-efficacy**

Hanging (Who, When and Where)

Capability or willingness to use nets

1. **Malaria information and services**

Access and affordability

# Key Informant Interviews (KIIs) and In-Depth Interviews (IDIs)

Education, Malaria history, Social roles, Salary or money income

1. **Ownership**

Do you have nets?

Does the net have holes?

Did you replace old nets?

1. **Use of ITNs**

Did you use nets yesterday? Why?

What is your motivation for using nets?

Do you use nets for purposes other than malaria prevention?

1. **Malaria risk perception**

Are you afraid of malaria? Why?

What causes malaria?

What can you do to reduce your risk?

1. **Perceived benefits of and barriers to ITN use**

Are there any good points or bad points?

Which do you prefer treated nets or non-treated nets?

1. **Self-efficacy**

Are you willing to use nets?

1. **Malaria information and services**

How do you get the information or tools you need for malaria prevention?
